# Treating locally advanced and metastatic cutaneous squamous cell carcinoma with anti-PD-1 drugs: Real-world experience in 72 patients

**DOI:** 10.1016/j.jdin.2023.06.013

**Published:** 2023-07-06

**Authors:** Robin M. Picavia, Jennifer DeSimone, Sekwon Jang, Chelsea Stahl

**Affiliations:** a University of Virginia School of Medicine, Charlottesville, Virginia; b INOVA Schar Cancer Institute Melanoma and Skin Oncology Center, Fairfax, Virginia

**Keywords:** clinical research, drug response, general dermatology, immunomodulators, medical dermatology, oncology

*To the Editor:* Cutaneous squamous cell carcinoma (cSCC) is highly curable when localized. In approximately 1% to 5% of cases, locoregional or distant metastases may occur, resulting in significant morbidity and 4000-8800 patient deaths annually in the United States.^1^ Current guidelines call for curative surgery and/or radiation therapy as first-line, which may result in prolonged and complicated recovery, disfigurement, and loss of function. Anti-programmed cell death protein 1 (PD-1) antibody drugs, Cemiplimab (Cem), and Pembrolizumab (Pem) are indicated for the treatment of locally advanced and metastatic cSCC. Here, we aim to share our real-world experience using anti-PD-1 agents in a large cohort of patients. We highlight our observation that single-agent immunotherapy is well-tolerated and effective, achieving durable complete responses in many patients with 4 or fewer total infusions.

This retrospective study analyzed 72 adult patients treated for locally advanced or metastatic cSCC at the INOVA Schar Cancer institute in Fairfax, VA. Evaluation of treatment response was performed using RECIST criteria with patients graded as having a complete response (CR), partial response (PR), stable disease (SD), or progressive disease (PD).^2^ Duration of response was defined as no evidence of disease on clinical exam and/or radiologic imaging and most recent follow up examination. Adverse events were also noted, including severity and specific event type.

Table I summarizes the results, which included 72 patients with locally advanced (*N* = 46) or metastatic (*N* = 26) cSCC treated with PD-1 inhibitor monotherapy between March 2017 and February 2023. Thirteen (18%) patients required 4 or fewer infusions for a complete response. Findings also included an overall response rate (ORR) of 72% (52/72) and a complete response rate (CR) of 50% (36/72).

**Table I:** Patient demographics, safety, and outcome data

Characteristic		No. of patients (%, *N* = 72)	
Male		57 (79)	
Median age (range), yr		81 (43-98)	
ECOG performance status∗			
0-2		63 (86)	
3-4		9 (13)	
Tumor characteristics			
Stage		Site	
Locally advanced	46 (64)	Lymph node	25 (35)
Metastatic	26 (36)	Head and neck	38 (53)
		Extremities	4 (6)
		Trunk	5 (7)
		Adverse Event no. (%)	
Any adverse events (AEs)		29 (40)	
No reported AEs		41 (57)	
Severity			
Mild (grade 1-2)		27	
Serious (grade 3-5)		7	
Mild adverse events		Severe adverse events	
Hypothyroidism	1	Diarrhea	1
Psoriasis flare	1	Elevated LFT’s	1
Rash	4	Rash	2
Fatigue	8	Arthralgia	1
Arthralgia	2	Myocarditis	1
Pneumonitis	4	Pneumonitis	1
Pruritis	6		
Diarrhea	1		
Best overall response no. (%)			
Complete response (CR)		36 (50)	
Partial response (PR)		12 (17)	
Stable disease (SD)		4 (6)	
Progression of disease (POD)		20 (28)	
Overall response rate†		52 (72)	
Patients with CR in ≤4 infusion		13 (18)	
Median no. of infusions (range)		6 (1-25)	
DOR‡, median (IQR), weeks		47 (29.5-94.25)	

Number in parenthesis represent average values unless otherwise specified.

∗ ECOG Performance grade at time of first infusion.

† ORR: Includes patients with PR, CR, and SD.

‡ DOR: Duration of response and includes values from CR, PR, and SD groups.

Previous studies of Pem in advanced, metastatic, or recurrent cSCC reported an ORR of 40.3%, with 12.6% reaching a CR.^3^ A phase II study of Cem on 78 patients with locally advanced cSCC reported an ORR of 44%, with 13% of patients achieving a CR.^4^ A similar study of 131 patients reported an ORR of 58%.^5^ We report our experience treating 72 advanced cSCC patients in our multidisciplinary hospital-based clinic. Our ORR was 72%, and the CR rate was 50%, considerably higher than previously reported. Excellent tolerability was observed overall.

Advanced cSCC patients are often elderly, with severe comorbidities and reduced functional capacity. We discovered that such patients are successfully treated with very few (≤4) total infusions, thus limiting treatment burden and health care environment exposure as the SARS-Cov2 pandemic lingers. These complex and fragile patients avoided surgical complications, loss of function, and permanent disfigurement, commonly encountered with current first-line surgery and radiation. We have learned to be thoughtful and collaborative among our multidisciplinary team and with our patients in identifying the optimal individual treatment course, considering both medical and procedural options. Limitations of this case series are lack of standardized controls, variations in dosing regimens and duration of therapy, and relatively short follow-up. Future studies examining the recurrence rates in advanced cSCC patients treated with anti-PD-1 monotherapy versus standard-of-care surgery plus or minus adjuvant radiation are necessary for correctly prognosticating and managing this population.

## Conflicts of interest

None disclosed.
